# Serological and Demographic Correlates of HBV DNA Detection Below the Limit of Quantification in Treated Chronic Hepatitis B and HBsAg-Negative Patients

**DOI:** 10.3390/biomedicines14061191

**Published:** 2026-05-25

**Authors:** Hasan Zeybek, Tugrul Hosbul

**Affiliations:** 1 Department of Medical Microbiology, Gulhane Training and Research Hospital, 06010 Ankara, Türkiye; 2 Department of Medical Microbiology, Gulhane Faculty of Medicine, University of Health Sciences, 06010 Ankara, Türkiye; tugrulhosbul@gmail.com

**Keywords:** hepatitis B virus, very low-level viremia, lower limit of quantification, risk factors, occult hepatitis B virus infection

## Abstract

**Objectives**: This study aimed to evaluate very low HBV DNA viral load below the limit of quantification and to identify correlational factors in different patient groups, including individuals with chronic hepatitis B (CHB), occult HBV infection (OBI), and others. **Methods**: We retrospectively analyzed 390 patients with very low-level viremia (VLLV). HBV DNA levels were measured in plasma samples using real-time quantitative PCR (qPCR). Serological markers were evaluated in serum samples using chemiluminescence microparticle immunoassay (CMIA). Demographic variables, HBV serological markers (anti-HBs, anti-HBe, anti-HBc), and DNA results were evaluated. **Results**: The study included 193 CHB patients with maintained virological suppression and 197 patients in the other group; of which, 60 patients had occult hepatitis B infection (HBV DNA positive, HBsAg negative) and 137 had no occult hepatitis B infection. Very low viral load was more common in men (53.3%) and in individuals aged ≥50 years (63.3%). In univariate analysis, OBI was associated with anti-HBe (odds ratio (OR) = 2.874, 95% CI: 1.255–6.579, *p* = 0.013), and anti-HBc seropositivity (OR = 5.750; 95% CI: 2.626–12.591, *p* < 0.001). In multivariate analysis, anti-HBe positivity and anti-HBc positivity were independently associated with OBI. Anti-HBs positivity was independently and inversely associated with OBI. **Conclusions**: In patients with VLLV cohort, anti-HBc and anti-HBe seropositivity were independently associated with detectable but unquantifiable HBV DNA. Although anti-HBe positivity reflects reduced viral replication, it does not indicate complete viral suppression and may be detected at very low viremia levels, especially in occult HBV infection. These findings highlight the complex interplay between viral replication dynamics and host immune responses across the VLLV spectrum, characterize the serological landscape associated with detectable but unquantifiable HBV DNA, and warrant validation in prospective studies.

## 1. Introduction

Hepatitis B virus (HBV) infection remains a major global health concern, affecting approximately 296 million people and causing nearly 1.1 million deaths annually due to HBV-related complications [1]. Despite the widespread implementation of universal vaccination, the incidence of new infections has decreased; however, chronic hepatitis B (CHB) continues to impose a significant burden on global healthcare systems [2]. Nevertheless, contemporary antiviral approaches predominantly employ nucleos(t)ide analogs (NAs), including entecavir (ETV) and tenofovir, which proficiently inhibit virus replication; however, a significant number of patients do not attain a complete virological response, resulting in the clinical occurrences of occult HBV infection (OBI) and chronic very low-level viremia (VLLV) [3,4,5]. The fundamental mechanism driving both OBI and VLLV is the enduring presence of covalently closed circular DNA (cccDNA) in the nucleus of infected hepatocytes [2,6]. Current nucleic acid therapeutics incorporate into viral DNA to inhibit replication but are incapable of immediately eradicating or silencing the persistent cccDNA reservoir [7]. Host immunological failure, which is characterized by insufficient T-cell and B-cell responses that fail to achieve complete viral clearance, exacerbates this persistence. Additionally, this pattern may exhibit a maintained virological suppression to antiviral treatment for CHB [8,9,10,11].

VLLV refers to a specific subset of low-level viremia where HBV DNA remains detectable at extremely low concentrations, typically defined as <20 IU/mL. Advances in PCR technology facilitates highly sensitive detection of HBV DNA, attaining a lower limit of detection (LOD) below 10 IU/mL [12]. The limit of detection is generally less than the limit of quantification (LOQ). Methodologically, VLLV and OBI management is intricately linked to a precise comprehension of the technical differentiation between the LOD and LOQ [5,13,14]. In diagnostic approaches, confusion in clinical practice between the LOD and LOQ may lead to the misdiagnosis of actual persistent viremia and OBI cases [15].

Currently, contemporary high-sensitivity PCR assays (LOD < 10 IU/mL) have demonstrated that in roughly 20% to 40% of patients undergoing antiviral therapy, the virus is not entirely eradicated, and very low-level replication persists [4,12]. Laboratory reports often classify results into three categories: “target not detected” (<LOD), “<LOQ” (detectable but not quantifiable), or a specific numerical value within the linear range. A major issue in clinical practice and research is utilizing the erroneous application of the LOQ value instead of the LOD. This variability frequently results in inconsistent outcomes across various studies and misinterpretation by laboratory specialists and clinicians, thereby influencing clinical decision-making. Intermittent positive PCR findings may occasionally occur above the detection limit (>LOD) but below the quantification limit (<LOQ). It is essential for identifying missed OBI diagnoses or diagnostic discrepancies and evaluating CHB progression risk in some cases under the therapy [9,16]. It is also crucial for assessing the risk of occult HBV infection (OBI) reactivation or transmission. This limitation restricts clinicians to determining the presence of HBV DNA in the patient’s blood, which may lead to misinterpretation of findings suggestive of OBI.

The objective of our study is to examine very low-level viremia and characterizing the serological and demographic correlates of HBV DNA detection below the LOQ within a cohort of treated CHB and HBsAg-negative patients for diagnostic and therapeutic monitoring, thereby establishing a foundation for a deeper comprehension of the significance of viral load between >LOD and <LOQ in clinical practice. Additionally, our study aims to reduce the potential misinterpretation of significant results between the detection limit and quantification limit, taking into account the diagnostic sensitivity of HBV DNA tests, and to examine the impact of these results on the OBI detection rate when assessed alongside anti-HBe and other serological markers.

## 2. Materials and Methods

### 2.1. Study Design, Measurements and Ethical Approval

This study retrospectively analyzed in patients who attended the infectious diseases, gastroenterology, and some other internal medicine clinics at Gulhane Training and Research Hospital in Ankara Province. Plasma samples sent for laboratory diagnosis and viral load assessment of Hepatitis B from January 2020 to October 2025 were incorporated into the retrospective investigation. The analysis encompassed factors such as gender, age, reason for visit/initial diagnosis, HBV serological markers, including HBsAg, anti-HBs, anti-HBe, anti-HBc and HBV DNA test results.

HBV DNA was quantified by real-time fluorescent quantitative polymerase chain reaction (qPCR), considering the analytical sensitivity and detection limits of the assay. Amplification was carried out on plasma samples using the Montania 4896 Real-Time PCR system (Anatolia Geneworks, Istanbul, Turkey), while serological markers were assessed in serum samples using the chemiluminescent microparticle immunoassay (CMIA) method on the Cobas e801 analyzer (Roche Diagnostics, Mannheim, Germany) and the Architect i2000sr system (Abbott, Chicago, IL, USA).

HBV serological assays acquired from using the CMIA method on Architect i2000sr and Cobas e801 instruments, HBsAb < 10 IU/mL, HBsAg < 1.00 S/CO, HBeAb > 1.00 S/CO were classified as negative. While HBcAb < 1.00 S/CO test results are evaluated as negative on the Architect system, on the Cobas e801 analyzer a S/CO value > 1.0 was regarded as positive due to the competitive binding process.

The limit of detection (LOD) by PROBIT at a 95% hit rate for this detection method was <6.75 IU/mL when analyzing 400 μL of plasma. The anticipated linear range of test kit HBV extended from the limit of quantification (LOQ) at 10 IU/mL in a 400 μL sample volume to the upper limit of quantitation (ULOQ) at 1.00 × 10^9^ IU/mL. All experimental procedures and analyses were conducted in strict accordance with the protocols, following the manufacturer’s instructions. The test results are presented in the following formats: target not detected (<LOD), target detected but unquantifiable (<LOQ), or HBV DNA identified within the linear range of LOQ < x < ULOQ.

**Figure 1:**
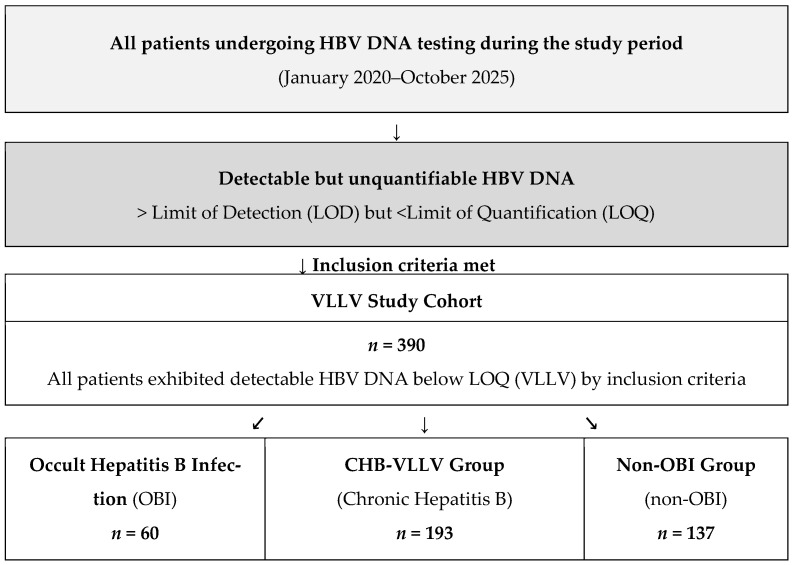
Flowchart of the participants. Subgroup classification was based on serological and clinical criteria applied within the VLLV (very-low level viremia) cohort.

We screened 390 patients with VLLV (Figure 1). The main requirements for inclusion were (i) being at least 18 years old; (ii) having chronic HBV infection under antiviral medication for years; and (iii) having biochemical abnormalities believed to be associated with hepatocellular diseases from the clinicians’ perspective. The exclusion criteria encompassed concurrent infection with viruses other than HBV, such as HIV and hepatitis C, and if data were unavailable.

The study was conducted in accordance with the Declaration of Helsinki, and the protocol was approved by the ethics committee at University of Health Sciences Gulhane Scientific Research (decision no: 2025-592) on 23 December 2025.

### 2.2. Description of OBI Diagnosis and CHB Group in the Study

Per the Taormina consensus statement, OBI is defined as the presence of HBV DNA in hepatic tissue, with absent or low-level HBV DNA in serum, in HBsAg-negative individuals [17]. Confirmatory diagnosis of OBI ideally requires intrahepatic HBV DNA assessment, which was not systematically available in this retrospective cohort. Accordingly, patients in this study were classified as operationally defined OBI based on HBsAg negativity and detectable serum HBV DNA below the LOQ, consistent with the serological component of the Taormina criteria. In this study, the findings of single high-sensitivity HBV DNA testing were analyzed independently for participants who underwent multiple tests. For the purposes of this study, the CHB group was classified as the maintained virological suppression due to the history of antiviral therapy screening routine follow-up.

### 2.3. Statistical Analysis

Statistical analyses were conducted utilizing GraphPad Prism program 10.6.1 (892) at a 95% confidence level. The Shapiro–Wilk test and visual methods (histogram/Q-Q plot) were used to check how continuous data were spread out. Variables that were regularly distributed were shown as mean ± SD, while variables that were not normally distributed were shown as median (IQR). For categorical variables, counts (*n*) or percentages (%) were applied, with comparisons conducted using Fisher’s exact test where expected cell counts were <5. Multivariable logistic regression was performed using a combined variable selection strategy: variables achieving *p* < 0.10 in univariate analysis were included as candidate variables, and three clinically established variables—age (≥50 vs. <50 years), sex, and anti-HBe positivity—were entered into the model irrespective of their univariate *p*-values using a forced entry approach, given their recognized relevance in HBV viral dynamics and NA-treated cohort analyses [12,18,19].

A multivariate logistic regression analysis was employed to assess the correlation between serological/demographic factors and OBI status within the VLLV cohort. Independent variables include anti-HBe (±), total anti-HBc (±), anti-HBs (±), age < 50 (years), age ≥ 50 (years), and gender (male/female). The results were given as ORs, 95% CIs, and *p*-values.

## 3. Results

### 3.1. Summary of Baseline Characteristics of the Patients

This retrospective study conducted from January 2020 to October 2025 comprised a total of 390 patients: 193 in the CHB group, 197 in the other group. The other group consist of 60 OBI (HBV DNA positive and HBsAg negative) and 137 non-OBI patients. CHB patients receiving antiviral treatment were classified as the maintained virological suppression. All results exhibited VLLV below the LOQ. Table 1 clearly shows the fundamental demographic attributes and serum virological marker of all participants for the CHB group with maintained virological suppression, as well as the OBI and non-OBI groups. The mean age of all CHB patients was 49.1 years, with 52.8% as male. Patients with VLLV were primarily male (*n* = 208; 53.3%) and 247 (63.3%) were aged 50 years and over. Likewise, the non-OBI group consisted mainly of males (*n* = 78; 56.9%); 82 (59.9%) were older than 50 years of age. OBI and non-OBI patient groups had various clinical features, such as previous or resolved HBV infection, cirrhosis, abnormal liver function tests of unidentified etiology, diabetes mellitus (DM), liver disease, hepatocellular carcinoma and high-level hepatic function chemistry parameters, which involved alanine aminotransferase (ALT), aspartate aminotransferase (AST), total bilirubin, gamma-glutamyl transpeptidase (GGT), alpha-fetoprotein (AFP), lactate dehydrogenase (LDH), and alkaline phosphatase (ALP).

### 3.2. Comparison of Specific Characteristics Within OBI and CHB Groups

The results of the univariate analysis indicated significant differences in anti-HBe and anti-HBc between OBI and non-OBI groups. Likewise, other parameters including sex, age ≥ 50 years, and anti-HBs status were calculated separately in univariate analysis (Table 2). The univariate analysis findings were incorporated into the multivariate logistic regression model to examine the correlational factors associated with OBI.

### 3.3. Variables of OBI and Non-OBI Groups

In the univariate analysis, we observed a significant correlation between the proportion of OBI cases and HBeAb seropositivity (odds ratio (OR) = 2.874, 95% confidence interval (CI): 1.255–6.579, *p* = 0.013), as well as HBcAb (OR = 5.750; 95% CI: 2.626–12.591, *p* < 0.001) cases, as detailed in Table 2. Furthermore, male sex, age ≥50 years, and HBsAb levels exhibited no correlation with the OBI classification with VLLV. In multivariable logistic regression analysis, anti-HBe and anti-HBc positivity remained independently associated with OBI (OR = 2.670, 95% CI: 1.100–6.490, *p* = 0.03; OR = 5.690, 95% CI: 2.550–12.720, *p* < 0.001 respectively). Anti-HBs positivity was inversely associated with OBI but sex was not independently correlated with it.

## 4. Discussion

This study examined the association between very low-level HBV viremia and serological markers, such as anti-HBe, anti-HBs, and total anti-HBc, alongside demographic characteristics in groups of OBI, non-OBI and CHB patients with maintained virological suppression. In addition our research underscores the challenges of very low-level viremia in CHB patients undergoing nucleoside analog therapy, as well as in cases of occult HBV infection and non-OBI patients accompanying the existence of other prevalent liver conditions performed by highly sensitive HBV DNA assay.

Several academic studies indicate that persistent very low viral replication occurs in around one-third of patients despite efficient antiviral treatment, and that the incorrect application of LOD/LOQ values in the HBsAg-negative cohort results in a substantial diagnostic deficiency in OBI diagnosis [15]. While the principal objective in HBV management is the total suppression of viral replication, both studies highlight that specific virological markers (including elevated HBsAg or HBeAg levels, anti-HBc and anti-HBe status) are significant risk factors for low-level viral persistence in both CHB and OBI patients, thus emphasizing the necessity for a more dynamic monitoring approach [12,20,21]. The mechanisms driving the development of VLLV are inadequately comprehended and are probably affected by many circumstances. Prior research indicates possible correlations with the cccDNA reservoir [7], host immunological variables, nucleoside analog resistance and constraints, in addition to patient compliance [12]. Nevertheless, several baseline clinical features and virological data may be associated with the presence of LLV. Kim et al. recognized baseline HBV DNA burden as a significant risk factor for LLV [22]. Within the cohort examined by Li et al. [23], the proportion of patients with baseline HBV DNA ≥ 6.0 log_10_ IU/mL was substantially greater in the LLV group (63.8%) than in the CVR group (45.1%). In our study, we did not assess the correlation between the presence of LLV and baseline DNA levels. Our analysis evaluated the association between the VLLV and age factor, HBV biomarkers such as anti-HBc, anti-HBe, and anti-HBs. We revealed that anti-HBc and anti-HBe seropositivity were independently associated with the OBI profile. Our findings are consistent with previous studies.

Improper use of the LOD for HBV DNA detection kits will substantially impact the detection rate of occult hepatitis B infection. Pronier et al. [24] conducted a study revealing that out of 52 OBI cases, only 23 cases (44.2%, 23/52) exhibited HBV DNA levels ≥ 10 IU/mL, utilizing the lower limit of quantification (LOQ = 10 IU/mL) as the diagnostic criterion. Conversely, 29 cases (55.8%) were identified (detection limit unspecified) but could be overlooked as the test results were below 10 IU/mL. Incorrectly using LOQ as LOD and interpreting results below LOQ as negative for HBV DNA could lead to diagnostic error of OBI, necessitating our focus in clinical practice. Our findings indicate that when the limit of quantification is erroneously applied as the limit of detection, HBV DNA results for all patient groups in our study were reported as ‘target not detected.’ This finding may be of limited interpretive impact for treated CHB patients, while remaining relevant for OBI identification, as virological success achieved under antiviral therapy, together with very low-level viremia positivity or a negative (‘target not detected’) result, may be considered clinically equivalent from the clinician’s perspective. However, accurate identification of OBI remains relevant for laboratory reporting accuracy, particularly in patients with serological evidence of prior HBV exposure, and in the presence of underlying diseases or other predisposing factors.

Within our study cohort of 390 VLLV patients, the proportion classified as operationally defined OBI was 15.3% (60/390) among all VLLV outpatients at the Gulhane Training and Research Hospital Medical Microbiology Department in Turkey. Among these cases, 85% of OBI (51 out of 60) were anti-HBc positive. When viewed from a different perspective among those who were anti-HBc positive, 13.07% (51 out of 390) exhibited OBI. In the anti-HBc-positive group, the proportion of OBI within the VLLV cohort is affected by the presence of anti-HBe and anti-HBs; notably, larger detection rates occur in anti-HBe-positive individuals, whereas lower rates are observed in anti-HBs-positive people. These findings indicate that anti-HBe serves as both a risk and a protective factor for OBI, as supported by the multivariate analysis. Individuals who are anti-HBe positive have a 2.67-fold higher relative risk of being diagnosed with OBI compared to those who are anti-HBe negative. In light of these findings, our serological findings suggest an association between anti-HBe positivity and OBI classification within this cohort, warranting further investigation in individuals with a history of HBV infection. In other words, our findings show an association between anti-HBe positivity and detectable HBV DNA below the LOQ in this cohort, consistent with the natural course of chronic HBV infection.

Previous studies indicate that low-level viral replication persists in a subset of chronic hepatitis B patients on extended antiviral therapy [4]. This persistence likely stems not only from treatment adherence but also from a complex interaction between viral characteristics and host immunological regulation, both of which could contribute to partial viral suppression. It should be noted that our study did not include a non-VLLV comparator group for CHB patients, as our cohort consisted exclusively of individuals who already exhibited the VLLV pattern (detectable HBV DNA below the LOQ). Consequently, we cannot estimate the proportion of CHB patients on antiviral therapy who exhibit a VLLV pattern nor can we predict the VLLV classification. Instead, we have focused on evaluating the serological landscape within this specific cohort. Considering that the dynamics of immunological maturation, antigen exposure, and potential immunological exhaustion likely affect the viral phenotype across different age groups [25], our study observes that anti-HBe and anti-HBc positivity increases in correlation with age. We believe these findings may be related to the influence of immune exhaustion and host factors. We believe these findings may be related to the influence of immune exhaustion and host factors. This study comprehensively evaluated the serovirological profile of patients with very low-level HBV viremia and determined that anti-HBe and anti-HBc positivity were independently associated with occult HBV infection (OBI). In the multivariable model, anti-HBs positivity was independently and inversely associated with OBI classification. Our findings suggest that anti-HBe positivity is associated with a lower likelihood of viral replication. However, the presence of anti-HBs does not necessarily indicate complete viral suppression. From a clinical perspective, further prospective studies are warranted to determine the clinical implications of VLLV in this patient population—especially those with persistently low-level replication—remains essential, even when favorable serological markers are present.

### Limitations

This study has several limitations. First, its retrospective design limits the ability to draw causal inferences. Second, the sample size of the OBI cohort exhibiting a very low-level viremia pattern was insufficient. Third, treatment conditions and duration may have influenced viral dynamics, and long-term clinical outcomes were not evaluated. Furthermore, individual-level data on specific antiviral agent allocation (ETV vs. TDF/TAF) and treatment adherence were not systematically captured in our retrospective database. Given the established differences in viral suppression dynamics between these agents [26], the absence of regimen stratification introduces unmeasured confounding that cannot be addressed within the present analytical framework. Fourth, quantitative measurements of anti-HBc and intrahepatic markers—which are particularly relevant to OBI—were not available. Fifth, systematic confirmatory retesting via repeat nucleic acid extraction or alternative assay platforms to exclude random detection near the LOD or pre-analytical contamination could not be performed for all samples in this retrospective setting. Finally, the findings related to the VLLV pattern were not all replicated with a new sample or re-test.

The present study should therefore be interpreted as exploratory and hypothesis generating; its findings identify serovirological associations that warrant formal testing in prospective cohort studies with pre-specified data collection protocols.

## 5. Conclusions

Among individuals within the VLLV cohort, anti-HBc and anti-HBe seropositivity were independently correlated with occult hepatitis B infection (OBI). Very low levels of HBV DNA in anti-HBe-positive patients suggest that this marker reflects an active immune response rather than complete viral eradication. Although anti-HBe seropositivity is generally associated with suppression of viral replication and transition to the immune control phase, it does not exclude the presence of persistent low-level viral activity, particularly in cases of OBI. Therefore, these findings suggest that anti-HBe status may inform future investigations in VLLV characterization, especially when interpreted alongside HBV DNA levels and other serological markers.

Anti-HBc positivity was strongly associated with operationally defined OBI within this cohort, supporting its potential utility in future prospective characterization studies. These findings warrant validation in prospective case–control studies designed to assess long-term clinical implications and host immune response in the VLLV spectrum, as well as support further investigation in larger prospective cohorts. The clinical implications of VLLV remain incompletely characterized and warrant further hypothesis-driven research. Advances in PCR technology facilitate the monitoring of DNA levels and enhance the identification of additional VLLV patterns. Due to the lack of comparative information about the advantages of either maintaining original treatment or altering treatment method for patients with VLLV, additional clinical studies are necessary to elucidate these alternatives.

## Figures and Tables

**Table 1:** Baseline characteristics of CHB, OBI, and non-OBI groups with VLLV under LOQ.

Variable	All Groups (*n* = 390)	CHB Group (*n* = 193)	OBI Group (*n* = 60)	Non-OBI Group (*n* = 137)
Age, years (mean ± sd)	50.9 ± 14.2	49.1 ± 13.7	55.6 ± 12.6	51.5 ± 14.7
<50 years, *n* (%)	143 (36.7)	72 (37.3)	16 (26.7)	55 (40.1)
≥50 years, *n* (%)	247 (63.3)	121 (62.7)	44 (73.3)	82 (59.9)
Gender				
Male, *n* (%)	208 (53.3)	102 (52.8)	28 (46.7)	78 (56.9)
Female, *n* (%)	182 (46.7)	91 (47.2)	32 (53.3)	59 (43.1)
HBcAb status				
Negative, *n* (%)	80 (20.5)	2 (1)	9 (15)	69 (50.4)
Positive, *n* (%)	310 (79.5)	191 (99)	51 (85)	68 (49.6)
HBeAb status				
Negative, *n* (%)	79 (20.3)	29 (15)	8 (13.3)	42 (30.7)
Positive, *n* (%)	311 (79.7)	164 (85)	52 (86.7)	95 (69.3)
HBsAb status				
Negative, *n* (%)	310 (79.5)	189 (97.9)	42 (70)	79 (57.7)
Positive, *n* (%)	80 (20.5)	4 (2.1)	18 (30)	58 (42.3)

Abbreviations: VLLV, very low-level viremia; LOQ, limit of quantification; HBsAb, hepatitis B surface antibody; HBeAb, hepatitis B e antibody; HBcAb, hepatitis B core antibody; CHB, chronic hepatitis B; OBI, occult HBV infection.

**Table 2:** Comparison of specific variables within OBI and non-OBI populations by logistic regression analysis.

Variables	Univariate Analysis		Multivariate Analysis	
	OR (95% CI)	*p*-Value	aOR (95% CI)	*p*-Value
Age (years) ≥50 vs. <50	1.845 (0.947–3.592)	0.072	1.580 (0.760–3.290)	0.222
Sex Male vs. Female	0.662 (0.360–1.218)	0.184	0.620 (0.320–1.220)	0.169
Anti-HBe Pos. vs. Neg.	2.874 (1.255–6.579)	0.013	2.670 (1.100–6.490)	0.030
Anti-HBc Pos. vs. Neg.	5.750 (2.626–12.591)	<0.001	5.690 (2.550–12.720)	<0.001
Anti-HBs Pos. vs. Neg.	0.584 (0.305–1.116)	0.103	0.470 (0.230–0.960)	0.037

Abbreviations: OR, odds ratio; aOR, adjusted odds ratio; CI, confidence interval; OBI, occult hepatitis B infection.

## Data Availability

The data can be found in the data repository of the Medical Microbiology Department—Gulhane Training and Research Hospital. If you wish to request access to the data, please contact us at hsnzeybek86@gmail.com.
